# Toxicogenomic assessment of liver responses following subchronic exposure to furan in Fischer F344 rats

**DOI:** 10.1007/s00204-015-1561-2

**Published:** 2015-07-21

**Authors:** Hongyan Dong, Santokh Gill, Ivan H. Curran, Andrew Williams, Byron Kuo, Michael G. Wade, Carole L. Yauk

**Affiliations:** Environmental Health Science and Research Bureau, Healthy Environments and Consumer Safety Branch, Health Canada, Ottawa, ON K1A 0K9 Canada; Bureau of Chemical Safety, Health Canada, Ottawa, ON K1A 0K9 Canada

**Keywords:** Low dose furan, Rat liver, Gender differences, Pathway benchmark dose, miRNA, Thryoid hormone

## Abstract

Furan is a widely used industrial chemical and a contaminant in heated foods. Chronic furan exposure causes cholangiocarcinoma and hepatocellular tumors in rats at doses of 2 mg/kg bw/day or greater, with gender differences in frequency and severity. The hepatic transcriptional alterations induced by low doses of furan (doses below those previously tested for induction of liver tumors) and the potential mechanisms underlying gender differences are largely unexplored. We used DNA microarrays to examine the global hepatic mRNA and microRNA transcriptional profiles of male and female rats exposed to 0, 0.03, 0.12, 0.5 or 2 mg/kg bw/day furan over 90 days. Marked gender differences in gene expression responses to furan were observed, with many more altered genes in exposed males than females, confirming the increased sensitivity of males even at the low doses. Pathway analysis supported that key events in furan-induced liver tumors in males include gene expression changes related to oxidative stress, apoptosis and inflammatory response, while pathway changes in females were consistent with primarily adaptive responses. Pathway benchmark doses (BMDs) were estimated and compared to relevant apical endpoints. Transcriptional pathway BMDs could only be examined in males. These median BMDs ranged from 0.08 to 1.43 mg/kg bw/day and approximated those derived from traditional histopathology. MiR-34a (a P53 target) was the only microRNA significantly increased at the 2 mg/kg bw/day, providing evidence to support the importance of apoptosis and cell proliferation in furan hepatotoxicity. Overall, this study demonstrates the use of transcriptional profiling to discern mode of action and mechanisms involved in gender differences.

**Electronic supplementary material:**

The online version of this article (doi:10.1007/s00204-015-1561-2) contains supplementary material, which is available to authorized users.

## Introduction

Furan (CAS No. 110-00-9) is a colorless, volatile and lipophilic compound that is used as an intermediate in the synthesis of many chemical and pharmaceutical agents, solvents, lacquers and resins. Furan has also been detected in a broad variety of foods, particularly coffee, canned meats and baby food, with levels exceeding 100 parts per billion (Bakhiya and Appel 2010). Furan in food is thought to be generated from the decomposition of carbohydrate, fatty acid and ascorbic acid during heat treatment processes such as cooking, canning and baking (Morehouse et al. 2008). In humans, furan has been detected in breast milk samples and in the breath of both smokers and passive smokers (NIH 2012).

Furan induces hepatocellular neoplasms and cholangiocarcinomas (CC) in both rats (F344) and mice (B6C3F1) in standardized 2-year carcinogenesis studies (NTP 1993). CC accounts for about 10–15 % of human primary hepatobiliary neoplasms worldwide (Chapman 1999), and CC-induced mortality has risen in the USA, Japan, Australia and Europe from 1979 to 1998 (Khan et al. 2002). Given that human exposure to furan is ubiquitous and the margin of exposure of furan compared to other rodent carcinogens is small (Carthew et al. 2010), the potential impacts of furan for human health are of concern.

Gender differences in the incidence of hepatocellular neoplasms in rats exposed to furan occurred in the 2-year cancer study, with increased frequency in male rats (NTP 1993). Benchmark dose (BMD) analysis of furan-induced hepatocellular carcinoma also indicated that male rats were more sensitive than females: estimated BMDs were 1.84 and 5.49 mg/kg bw in male rats and female rats, respectively (Carthew et al. 2010). Moreover, male rats exposed to 30 mg/kg bw furan for only 3 months from birth developed CC by 9 or 15 months of age (Maronpot et al. 1991).

Evidence in support of both genotoxic and non-genotoxic (hepatotoxicity and regenerative hepatocyte proliferation) modes of action (MoA) for furan toxicity have been reported (Hickling et al. 2010). Furan is negative for the induction of gene mutations in *Salmonella typhimurium* strains TA1535 and TA1537, and equivocal in strains TA98 and TA100 (Lee et al. 1994; Ronto et al. 1992; NTP 1993). Furan also yields inconsistent genotoxicity results in eukaryotic cells: (a) it is negative for the induction of sex-linked recessive lethal mutations in germ cells of male *Drosophila melanogaster* in vivo, sister chromatid exchanges in B6C3F1 mouse bone marrow cells in vivo and unscheduled DNA synthesis in hepatocytes of mice and rats in vivo; and (b) it is positive for the induction of trifluorothymidine resistance in mouse L5178Y lymphoma cells, sister chromatid exchanges in Chinese hamster ovary cells and chromosomal aberrations in B6C3F1 mouse bone marrow cells in vivo (Wilson et al. 1992; NTP 1993). Evaluation in rat livers in vivo suggests that genotoxicity from furan exposures may only occur at relative high doses via an indirect mechanism (Ding et al. 2012; McDaniel et al. 2012). Epigenetic alterations (DNA methylation and histone changes) are reported after 180 days of exposure to furan (Conti et al. 2014). Furan induces hepatotoxicity and proliferation at 0.1 mg/kg bw (Mally et al. 2010; Moser et al. 2009; Wilson et al. 1992). The primary MoA of furan-induced CC at carcinogenic doses is thus proposed to be oxidative stress, cytotoxicity and increased hepatocyte proliferation (Ding et al. 2012; Hickling et al. 2010). Although existing evidence supports that cell cycle perturbation and apoptosis are also involved in the MoA of furan at low doses (Chen et al. 2010), confirming this finding and investigating whether other MoAs are relevant at lower doses and occur to a similar extent in both sexes is required to provide critical information for human health risk assessment of furan exposure.

The MoA of furan in the livers of female mice (note: female mice were analyzed because of high spontaneous rates of liver tumors in male mice) exposed to both carcinogenic and non-carcinogenic doses for 21 days has previously been explored using gene expression profiling (Jackson et al. 2014). Marked changes in gene expression pathways involved in cytotoxicity, oxidative stress, inflammatory response and proliferation were observed, which is consistent with previously published histological data in other studies (Gill et al. 2011; Moser et al. 2009). The results demonstrate a clear distinction between adaptive low-dose responses (cytoprotective effects invoked through balanced activation of proliferative signaling and regeneration, sustaining a healthy liver) and adverse responses at high doses (overt cytotoxicity/proliferation in parallel with increases in pathways associated with pre-cancer and cancer cells). The results were an effective demonstration of the use of toxicogenomic data in elucidating the MoA and BMDs for furan oral exposures. A study conducted in male and female rats exposed to much lower doses of furan observed mild hepatic histological lesions at doses ≥0.12 mg/kg bw/day for 90 days (Gill et al. 2010). Clear gender differences were also observed in response to furan, with male rats exhibiting increased response over female rats for frequency of biliary hyperplasia and alterations of serum glucose, triglycerides, magnesium and thyroid hormones (TH). The application of whole genome transcriptional profiling (including non-coding RNAs) to explore the mechanism(s) and the influence of sex underlying furan-induced hepatotoxicity is an important next step in understanding the risk posed by human furan exposure.

The present study examines genomewide transcriptional responses in livers from our previous study of male and female F344 rats (Gill et al. 2010) exposed to furan by oral gavage over a 90-day period. Our analyses focused on the known lowest carcinogenic dose (2 mg/kg bw/day) and three lower doses. Livers from male and female rats from each dose were subjected to microarray analyses of mRNA and microRNA (miRNA) levels to assess global transcriptional changes. The transcriptional changes were directly related to various previously described phenotypic anchors to propose a mechanistically relevant transcriptional dose–response model. Gene expression changes were used to establish pathway BMDs to explore gender- and endpoint-specific responses for key events leading to furan-induced hepatotoxicity.

## Materials and methods

### Animals and exposures

Seven- to eight-week-old male or female F344 rats were exposed daily, 5 days per week, to furan in corn oil at doses of 0, 0.03, 0.12, 0.5 or 2 mg/kg/day by oral gavage for 90 days. Animal handling and treatment procedures were conducted according to the Guidelines of the Canadian Council of Animal Care and were approved by the Health Canada Animal Care Committee (Ottawa, ON, Canada). The full experimental details for this study, including histopathological and biochemical analyses, are reported elsewhere (Gill et al. 2010). Liver samples were collected 24 h after the last exposure, flash-frozen and stored at −80 °C.

### RNA extraction

Total RNA was extracted from frozen liver tissue of five male and five female rats from each dose group with *mir*Vana miRNA isolation kits (Ambion, Inc., Foster city, CA, USA) according to the manufacturer’s instructions. RNA integrity was determined using an Agilent 2100 Bioanalyzer (Agilent Technologies Inc., Mississauga, ON, Canada), and only high-quality RNAs (RNA integrity number ≥8.0) were used for microarray analysis of gene and miRNA expression.

### Microarray analysis of gene expression

A total of 50 RNA samples (five female and five male rats per dose group, five dose groups total) were labeled with cyanine 5-CTP using low-input quick amp labelling kits (Agilent Technologies Inc.) following the manufacturer’s instruction. Universal rat reference total RNA (Agilent Technologies Inc.) was labeled with cyanine 3-CTP. Cy5-sample cRNA and Cy3-reference cRNA were hybridized to Agilent G4853A SurePrint G3 Rat GE 8 × 60 K microarrays (Agilent Technologies Inc.) at 65 °C overnight with Agilent hybridization solution. Slides were washed and scanned on an Agilent G2505B microarray scanner at 5 μm resolution, and the data were acquired with Agilent Feature Extraction software version 10.7.3.1. One microarray from the male group failed standard quality checks and was eliminated from further analyses.

Raw signal intensity data were processed using *R* as described previously for data normalization, Fs calculation and *p* value adjustment with the false discovery rate (FDR, Paquette et al. 2011). The statistical model included gender and treatment as main factors as well as gender by treatment interactions. Genes were deemed to be ‘differentially expressed’ if either treatment or gender effects had a FDR *p* ≤ 0.05. The least squares mean was used to estimate the fold change (FC) for each pairwise comparison of interest (control versus each of the four dose groups, Goodnight and Harvey 1997; Searle et al. 1980). To explore gender differences in gene expression, we identified significantly changed genes in each dose group compared to control within each gender, followed by statistical analyses to determine the effect of gender. Cluster analysis was conducted with all significantly changed genes in all groups using GeneSpring GX 7.3 (Agilent Technologies Inc.). All data are available through the gene expression omnibus (GEO) website, accession number: GSE62808.

### qRT-PCR analysis of gene expression

Total RNA was reverse transcribed into cDNA using SuperScript III (Invitrogen, Burlington, ON, Canada). Quantitative PCR was performed in duplicate for each sample. A CFX96 real-time PCR detection system (Bio-Rad Laboratories, Mississauga, ON, Canada) was employed to detect SYBR green incorporation into amplified product. Gene expression levels were normalized to the housekeeping gene *Hprt*, which, based on microarray analysis, was unaffected by furan exposure. PCR efficiency was examined using the standard curve for each gene. Primer specificity was verified with a melting curve. OpenStat (http://www.statpages.org/miller/openstat/, a statistical program written by William G. Miller) was used for ANOVA of multiple groups, while the Fisher’s least significant difference test was used to detect significant differences (*p* < 0.05) between control and a given dose group.

### Pathways analysis

Enriched functional effects were identified by analyzing genes with FDR *p* ≤ 0.05 and absolute FC ≥ 1.5 relative to control in male and female rats exposed to furan at 2 mg/kg bw/day in Ingenuity Pathway Analysis (IPA, Ingenuity System, Inc., Redwood City, CA). We also applied IPA to the genes showing gender differences in expression at 2 mg/kg bw/day. Identification of canonical pathways within the data was determined by the enrichment of genes associated with pathways among the group of altered genes. Significance of the associated pathways was determined by IPA using a Fisher’s exact test (deemed significant if *p* ≤ 0.05). IPA predicted upstream modulators (including transcription factors, cytokines and chemical exposures), and toxicity functions were explored to identify potential regulators that might be consistent with the altered patterns of transcription observed. Statistical significance for the potential upstream modulators and toxicity functions were expressed as a *Z* score, where significant activation or association was inferred from a *Z* score ≥2, and significant inhibition or inverse associations from *Z* score ≤−2.

Functional annotation clustering to look at broad trends in expression patterns in males versus females was conducted using DAVID bioinformatics response 6.7 (Huang da et al. 2009a, b). The analysis was applied to genes that were significantly different in control males versus control females (FDR *p* ≤ 0.05 and absolute FC ≥ 1.5).

### Similarity to disease states or toxicity of other substances

Genes observed to be significantly altered (FDR *p* ≤ 0.05 and absolute FC ≥ 1.5) in either male or female rats from the 2 mg/kg bw/day dose group were uploaded into NextBio (http://nextbio.com) to identify disease states and/or chemicals with similar gene expression profiles using curated datasets. FC values (vs. control) were converted to ranks across the entire dataset and compared pairwise to curated datasets to generate a percent similarity metric.

### Determination of BMD for apical endpoints

The BMD (the estimated dose that caused a 10 % or 1 SD (standard deviation) change in observed responses) and the 95 % lower confidence limit (BMDL) for the apical endpoints of furan exposure observed in the rats used in the current study (Gill et al. 2010) were calculated using EPA BMDS 2.5 (Davis et al. 2011). Briefly, continuous dose–response models (Hill, polynomial, linear, power and exponential) and dichotomous models (gamma, logistic, loglogistic, logprobit, multistage, multistage-cancer, probit, weibull and quantal-linear) were applied for each endpoint, and BMDs were decided based on goodness-of-fit *p* value ≥0.1, absolute scaled residual ≤2 and visual curve inspection. When more than one model was suitable, the one with the lowest Akaike’s information criterion (AIC) was selected.

### Transcriptional BMD analysis

BMDExpress version 1.4.1(Yang et al. 2007) was used to calculate transcriptional BMDs and BMDLs. Briefly, genes observed to be significantly altered (ANOVA *p* ≤ 0.05 filtered) by any dose of furan exposure in either males or females were input into BMDExpress and evaluated by five models: Hill, power, linear 1°, polynomial 2° and 3°. For each probe, the best-fitting model was selected based on: (1) a nested Chi-square cutoff value of 0.05 to select between linear and polynomial models; (2) the lowest AIC to select between Hill and power models; and (3) a goodness-of-fit *p* value ≥0.1. Other parameters used included power restriction of ≥1, maximum iteration of 250, confidence level of 0.95 and a benchmark response (BMR) of 1.349 (the number of standard deviation defining the BMD, Yang et al. 2007). A Hill model was flagged if the “*k*” parameter was >1/3 of the lowest dose. The next best model with goodness-of-fit *p* value ≥0.1 was selected in place of a Hill model. If no other model had a goodness-of-fit *p* value ≥0.1, the Hill model was used and modified to 0.5 of the lowest BMD value. The resulting BMD datasets were mapped to IPA pathways (downloaded on April 24, 2014) to obtain functional classifications. Not all genes could be modeled, only BMDs that were less than the highest exposure dose and that had a goodness-of-fit *p* value ≥0.1 were included in subsequent analyses.

### miRNA microarray analysis

To explore the effects of furan on miRNA transcription, total RNAs from 16 liver samples (four males and four females from control and 2 mg/kg bw/day dose groups) were processed using the miRNA Complete Labelling and Hybridization kit (Agilent Technologies Inc.). Labeled RNA was hybridized on 8 × 15 K Agilent rat miRNA microarray slides. Arrays were scanned using an Agilent G2505B scanner (5 μm resolution). Feature extraction (version 10.7.3.1, Agilent Tech. Inc.) was used to acquire the fluorescence intensity of each probe.

The quality of the microarray data was evaluated using Agilent Feature extraction quality control metrics. Data were normalized in R using cyclic-lowess (Bolstad et al. 2003). Ratio–intensity plots, boxplots and cluster analyses were used to identify potential outliers. All samples passed the quality control tests and were used for subsequent analyses. Statistically significantly altered miRNAs were identified as described previously for gene expression. All data are available through the gene expression omnibus (GEO) website, accession number: GSE62807.

### qRT-PCR analysis of differentially regulated miRNAs

The miScript PCR system (Qiagen, Mississauga, ON, Canada) was used to examine the expression of miRNAs. Briefly, 1 μg of total RNA was used for polyadenylation of mature miRNAs and reverse transcription into cDNA using oligodT primers with a universal tag. RT-PCR was performed in duplicate for each sample using a primer complementary to the universal tag and a miScript primer assay specific to each miRNA. RNU6B was used to normalize the expression of the target miRNAs. FC was calculated using the $2^{{ - \Delta \Delta C_{{\text{t}}} }}$ method (Livak and Schmittgen 2001), and a student’s *t* test was used for statistical significance.

## Results

### General effects of furan on gene expression in rat livers

**Fig. 1:**
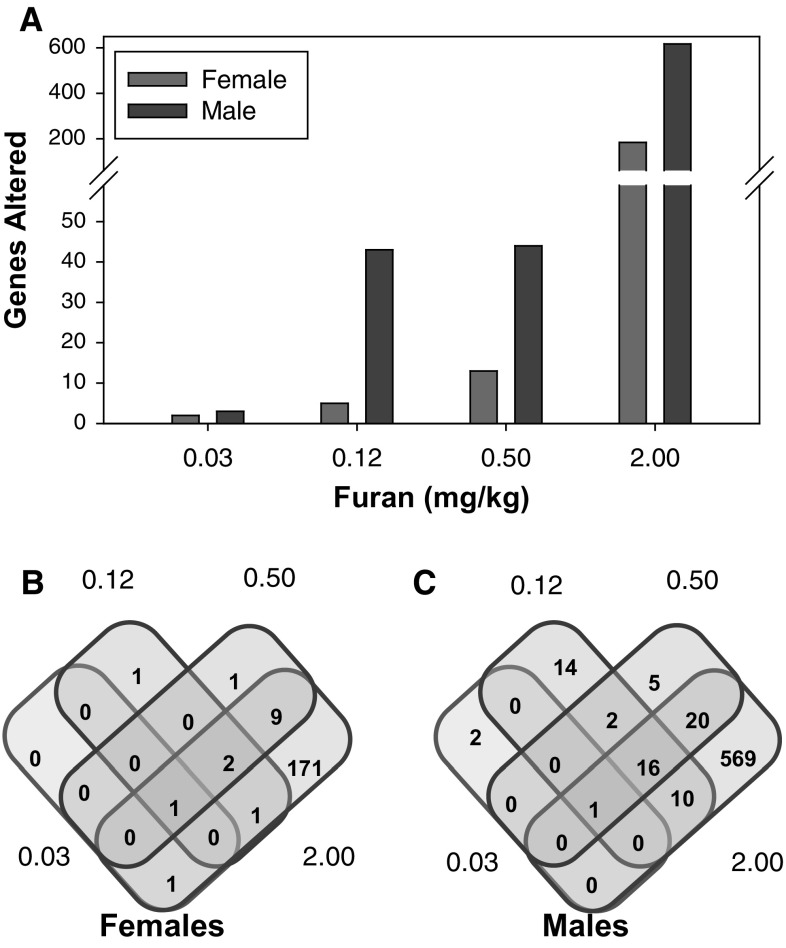
Number of significantly (FDR *p* ≤ 0.05) changed gene with absolute FC ≥ 1.5. **a** Dose-dependent increases in the number of genes that were differentially expressed in both genders relative to vehicle controls for increasing doses of furan were observed. **b** Venn diagrams showing the overlap of furan-responsive genes in each dose group in female rats. **c** Venn diagrams showing the overlap of furan-responsive genes in each dose group in male rats

The number of hepatic transcripts significantly (absolute FC > 1.5; FDR *p* ≤ 0.05) altered by furan exposure in rats increased with dose in both genders (Fig. 1a). FCs ranged from +10.52 to −5.93 for furan-treated relative to control rats. In female rats, genes that were affected in the lower-dose groups were generally affected in one or more of the higher-dose groups (Fig. 1b). In male rats, there were more responsive genes overall, and an increase in the number of genes that were uniquely affected within a dose group (Fig. 1c). The full lists of significantly altered genes (absolute FC ≥ 1.5 and FDR *p* ≤ 0.05) in females and males are shown in supplementary Table_S1 and Table_S2, respectively. The vast majority of gene expression changes were observed in the highest dose group, and of these, the majority were unique to the highest dose. Indeed, ~93 % of genes in both females (171/184) and males (569/617) were unique to the highest dose, indicating a clear transition in the nature of the transcriptional response to the 2 mg/kg/day dose of furan treatment.

Five genes were selected to validate microarray results using qRT-PCR (Table 1). These genes either had high FCs in male and/or female rats (*Abcb1b*, *Cyp3a62* and *Akr7a3*) or in the case of *Pparα* and *Fabp7* were related to the most affected pathway (LPS/IL-1-mediated inhibition of RXR function, Table 2). The qRT-PCR results were highly consistent with those from DNA microarrays for all five genes. The only exception was a detected increase in the expression of *Pparα* in the highest dose group of females by qRT-PCR that was missed by DNA microarray analysis (Table 1).

**Table 1:** Fold changes for select genes analyzed by microarray or real time RT-PCR

Furan (mg/kg bw/day)	*Akr7a3*		*Abcb1b*		*Cyp3a62*		*Pparα*		*Fabp7*	
	Female	Male	Female	Male	Female	Male	Female	Male	Female	Male
Microarray										
0.03	1.08	1.31	1.26	−1.04	1.12	1.41	1.3	1.42	−1.14	1.06
0.12	1.05	1.12	1.42	1	1.25	1.12	1.43	**1.76**	−1.11	1.06
0.5	1.36	**2.14**	**2.32**	**2.02**	1.3	**1.61**	1.42	**1.7**	**−1.52**	−1.15
2	**2.88**	**7.05**	**5.76**	**5.74**	**3.01**	**3.15**	1.32	**1.84**	**−2.07**	**−2.27**
RT-PCR										
0.03	1.61	2.18	2.26	1.41	1.05	1.03	1.51	1.54	1.14	1.25
0.12	1.96	0.76	2.09	0.67	1.12	1.60	1.52	1.14	1.22	**−2.97**
0.5	0.81	3.34	2.66	10.23	**1.85**	1.70	0.95	2.64	**−3.05**	**−1.54**
2	**3.82**	**16.65**	**40.82**	**35.11**	**3.07**	**3.49**	**2.48**	**3.27**	**−2.76**	**−3.22**

Bold font indicates *p* < 0.05 compared to control

**Table 2:** Canonical pathways affected by furan at 2 mg/kg bw/day in males and females

Female			Male		
Ingenuity canonical pathways	−log (*p* value)	Ratio	Ingenuity canonical pathways	−log (*p* value)	Ratio
Glutathione-mediated detoxification	2.99	0.07	**LPS/IL-1-mediated inhibition of RXR function**	11	0.114
**LPS/IL-1-mediated inhibition of RXR function**	2.97	0.03	**Acute phase response signaling**	8.25	0.116
**NRF2-mediated oxidative stress response**	2.73	0.03	Nicotine degradation II	6.23	0.129
Hepatic fibrosis/hepatic stellate cell activation	2.49	0.03	LXR/RXR activation	6.05	0.108
**Aryl hydrocarbon receptor signaling**	2.48	0.03	Coagulation system	5.49	0.211
**Histamine degradation**	2.42	0.07	Bupropion degradation	5.39	0.212
**Fatty acid α oxidation**	2.24	0.09	Acetone degradation I (to methylglyoxal)	5.27	0.194
**Oxidative ethanol degradation III**	2.24	0.05	Nicotine degradation III	5.05	0.123
**Putrescine degradation III**	2.19	0.07	PXR/RXR activation	4.95	0.109
2-Amino-3-carboxymuconate semialdehyde degradation to glutaryl-CoA	2.15	0.09	Melatonin degradation I	4.84	0.136
Methylglyoxal degradation VI	2.15	0.08	Xenobiotic metabolism signaling	4.82	0.073
**Tryptophan degradation X** (Mammalian, via Tryptamine)	2.14	0.07	FXR/RXR activation	4.72	0.1
**Ethanol degradation IV**	2.14	0.07	Complement system	4.64	0.2
**Acute phase response signaling**	2.13	0.03	Superpathway of melatonin Degradation	4.52	0.111
cardiomyocyte differentiation via BMP receptors	2.10	0.09	Methylglyoxal degradation III	4.48	0.227
**Granulocyte adhesion and diapedesis**	2.08	0.03	Estrogen biosynthesis	4.22	0.143
RAR activation	2.05	0.03	**Tryptophan degradation X** (mammalian, via tryptamine)	4.05	0.172
			Ethanol degradation II	3.66	0.14
			Bile acid biosynthesis, neutral pathway	3.52	0.069
			Noradrenaline and adrenaline degradation	3.44	0.113
			Dopamine degradation	3.42	0.132
			Extrinsic prothrombin activation pathway	3.14	0.182
			**Oxidative ethanol degradation III**	3.14	0.1
			Intrinsic prothrombin activation pathway	3.09	0.135
			**Ethanol degradation IV**	2.93	0.138
			Serotonin degradation	2.92	0.09
			**Aryl hydrocarbon receptor signaling**	2.84	0.064
			Retinol biosynthesis	2.64	0.111
			**Granulocyte adhesion and diapedesis**	2.57	0.066
			**NRF2-mediated oxidative stress response**	2.45	0.062
			Inhibition of matrix metalloproteases	2.43	0.125
			Thyroid cancer signaling	2.38	0.114
			Serine biosynthesis	2.38	0.154
			d-Glucuronate degradation I	2.38	0.143
			EIF2 signaling	2.37	0.06
			**Histamine degradation**	2.34	0.103
			Atherosclerosis signaling	2.24	0.065
			**Fatty Acid α oxidation**	2.08	0.13
			**Putrescine degradation III**	2	0.1
			Proline biosynthesis II (from arginine)	2	0.1
			Superpathway of serine and glycine biosynthesis I	2	0.111
			Arginine degradation VI (arginase 2 pathway)	2	0.125
			Superoxide radicals degradation	2	0.25
			Tyrosine degradation I	2	0.133
			Bladder cancer signaling	2	0.072

−log (*p* value) cutoff at 2, bold font indicates common pathways between males and females, ratio indicates the number of significant genes to the total number of genes in the pathway

### Pathways affected by furan exposure in male and female rats

Pathway analysis was conducted on genes with FDR *p* ≤ 0.05 and absolute FC ≥ 1.5 in males or females separately. With a pathway enrichment cutoff value of 2 [−log (*p* value)], only four (or fewer) canonical pathways were affected in the low-dose groups (data not shown). In females and males, 17 and 45 pathways, respectively, were affected by 2 mg/kg bw/day furan (Table 2); 65 % of the affected pathways in females were also significantly perturbed in males, including the LPS/IL-1-mediated inhibition of RXR and NRF2-mediated oxidative stress response pathways. Significant enrichment of biological functions, toxicological pathways or upstream activators with *Z* score ≥2 or ≤−2 and *p* < 0.05 only occurred in the highest dose groups. The larger number of affected genes in males at this dose resulted in more enriched molecular pathways/functions in males than females (Table 3). Male rat livers exposed to 2 mg/kg bw/day showed significant enrichment in 74 biological functions primarily related to inflammation and metabolism (supplementary Table_S3). In contrast, there was no significantly enriched biological function in females. Six toxicological pathways were enriched in males in the 2 mg/kg bw/day dose group; most of these were related to liver function (supplementary Table_S4).

**Table 3:** Enriched pathways, functions and activators in the differentially expressed gene lists from male and female rats exposed to 2 mg/kg bw/day furan

Categories	Male		Female	
	Increase	Decrease	Increase	Decrease
Biological functions	18 (liver injury and inflammation, cell death)	56 (lipid metabolism, hormone oxidation)	0	0
Toxicological functions	4 (liver injury and inflammation, cell death)	2 (liver hemorrhaging)	0	0
Upstream activators	27	25	5 (2*)	6 (1*)

Cut off vale: activation *Z* score ≥2 or ≤−2

* Number of elements common with male

Analysis of potential upstream regulators revealed 27 upstream modulators that were activated and 25 that were inhibited in males (supplementary Table_S5). These could be classified into various groups: chemicals and drugs, reagents, transcriptional regulators and endogenous hormones. Most of these were related to regulation of apoptosis, proliferation and cancer processes. Expression profiles were indicative of activation of five and inhibition of six upstream modulators in females (supplementary Table_S5). Only a few of upstream modulators were in common between the genders (RBPJ, CREB1 and 1,2-dithiol-3-thione; these are all involved in cell proliferation and apoptosis). Overall, pathway analysis indicated inflammatory and oxidative stress response, metabolism and cell proliferation pathways play important roles in the toxicity induced by furan in both genders. The pathway analysis further showed these processes and regulatory pathways were more perturbed in livers of male rats than female rats following furan exposure.

### Prediction of potential disease outcomes and chemicals with similar toxicity and pathways

Significantly changed genes with absolute FC ≥ 1.5 in male and female rats treated with 2 mg/kg bw/day furan were analyzed using NextBio (http://nextbio.com) to identify similarities in the expression profiles with curated studies. Phenotypes with scores >70 and at least three studies available for the disease model (only liver tissue in rodents was included) were considered. Disease models with similar gene expression profiles to furan-treated rats are shown in Table 4. Although all of the disease models identified as correlated with gene expression profiles in females were also correlated with males, these models were negatively correlated in females and positively correlated in males. This may indicate adaptive responses occurring in females versus adverse effects in males at this dose. The gene expression profile of the high-dose male rats was highly positively correlated with liver cancer, exhibiting the highest overall score as calculated from 55 similar studies. These studies included transgenic myc-driven liver tumors (GSE28198, 2011) and intrahepatic cholangiocarcinomas formed by inoculation of malignant rat cholangiocytes into liver (Dumur et al. 2010). Liver regeneration, injury of liver, inflammatory disease and cirrhosis were also highly positively correlated with furan treatment in male rats. Overall, these results are consistent with the hypothesis that furan induces liver injury, inflammation, regeneration and cancer in male rats.

**Table 4:** Diseases with similar gene expression profiles to rats treated with 2 mg/kg bw/day furan

Female				Male			
Phenotype score	Phenotype	# Studies	*R*	Phenotype score	Phenotype	# Studies	*R*
72.50	***High fat diet***	20	n	99.03	Liver cancer	55	p
70.04	***Diabetes mellitus***	32	n	89.80	***High fat diet***	30	p
69.98	***Disorder of endocrine pancreas***	33	n	85.17	Disease due to trypanosomatidae	6	p
69.42	***Injury of liver***	5	n	84.96	***Injury of liver***	3	p
				82.24	Inflammatory disease of liver	14	p
				79.16	Disease due to rotavirus	3	p
				78.19	Infection due to enterobacteriaceae	18	p
				77.43	Cirrhosis of liver	11	p
				77.19	Liver regeneration	4	p
				75.81	Hepatic fibrosis	11	p
				74.62	Deficiency state	22	p
				73.90	Disease due to adenovirus	3	p
				73.29	***Diabetes mellitus***	48	p
				73.28	***Disorder of endocrine pancreas***	50	p
				71.09	Bacterial infectious disease	8	p

Bold and italics font indicates common disease models between males and females

*R* refers to correlation. *n* indicates negative correlation, *p* indicates positive correlation

Only rodent liver tissues were included in this analysis

# Studies indicates the number of studies included in calculating phenotypes score

NextBio was also used to explore the similarity of gene expression profiles in rat livers treated with furan in the current study with other chemicals in the curated database. Using a cutoff score ≥80 and a requirement of at least three studies per chemical, we identified the chemicals inducing similar toxicity profiles to furan in male and female rats (supplementary Table_S6). The top five chemicals with the most similar profiles to furan-exposed male rats in our study are thioacetamide (TAA), nafenopin, methapyrilene (MP), mestranol and chloroform; these chemicals are all non-genotoxic carcinogens. The non-genotoxic carcinogens carbon tetrachloride (CCl4) and bromobenzene (BBZ) also induce similar gene expression profiles to furan treatment in male rats.

### Transcriptional BMD and apical endpoint BMD analysis

BMDExpress was used to establish transcriptional BMDs for furan. The responses were grouped using IPA pathways. BMD filtering included (1) pathways had to contain at least 10 genes; (2) at least five genes in the pathways had to exhibit a BMD ≤ 2 mg/kg bw/day and goodness-of-fit *p* value ≥0.1, as well as the percentage of genes modeled in the pathways had to account for at least 3 % of the pathway’s genes (however, we included small pathways if the number of genes that were significant and could be modeled accounted for at least 20 % of the pathway); and (3) pathways were excluded if the Hill model was the only model used for all of the genes within a pathway. There were 56 pathways that met all of the above criteria in males (supplementary Table_S7). The most sensitive pathway (i.e., lowest BMD) was MAPK signaling with a median BMD 0.08 mg/kg bw/day. No pathway passed the stringent filtering criteria in females. However, BMDs for 16 pathways were produced in females (supplementary Table_S7) when we loosened the stringency for the second criteria, instead requiring at least two genes or 1 % of the pathway affected. Half of these pathways were modeled in males too. The use of different filtering criteria to derive the pathway BMDs in males and females means the BMDs are not directly comparable, but provides a general view of the BMDs for females.

BMDs for apical endpoints (from our previous data, Gill et al. 2010) were calculated using EPA BMDS2.5. These were aligned to pathways that were representative of the apical endpoints. Apical and transcriptional BMDs were very close for liver cell damage and apoptosis, hepatocyte inflammation and biliary tract effects in male rats (Table 5). In females, apical endpoint BMDs for cholangiofibrosis and biliary tract hyperplasia were >2 mg/kg bw/day (supplementary Table_S8), while apical endpoint BMDs for Kupffer cell pigmentation and hepatocyte apoptosis were 0.11–0.20 mg/kg bw/day. As shown in supplementary Table_S7, we did not find any transcriptional BMD that were directly related to cell cycle regulation and apoptosis in females. Transcriptional and apical endpoint BMDs were not similar in females as there was an insufficient response to furan exposure at the doses used in current study.

**Table 5:** Comparison of apical and transcriptional BMDs in furan-treated males

Pathways	Transcriptional BMD(L)s			Apical BMD(L)s		
	# of genes modeled (total # of genes in pathway)	BMD median (mg/kg bw)	BMDL median (mg/kg bw)	Apical endpoints	BMD (mg/kg bw)	BMDL (mg/kg bw)
*Cell damage and apoptosis*						
p38 MAPK signaling	5 (108)	0.08	0.05	Kupffer cells pigmentation	0.03	0.02
ERK/MAPK signaling	6 (176)	0.17	0.04	Hepatocytes apoptosis	0.03	0.02
*NRF2*-*mediated oxidative stress response*	8 (169)	0.62	0.47			
*Inflammation*						
LPS/IL-1-mediated inhibition of RXR function	15 (186)	1.1	0.69	ALP	1.49	1.29
Acute phase response signaling	11 (157)	1.22	0.83			
*Biliary tract damage*						
Hepatic cholestasis	5 (126)	1.10	0.77	Biliary tract hyperplasia	1.45	0.54
FXR/RXR Activation	6 (79)	1.38	0.91	Biliary tract cholangiofibrosis	1.84	1.02
Bile acid biosynthesis, neutral pathway	2 (10)	1.43	0.94			

“# of genes modeled” indicates the number of genes that were significant by ANOVA and could be modeled (i.e., filtered to include genes with BMDs less than the highest dose of 2 mg/kg bw and *p* value of fit ≥0.1)

“Total # of genes in pathway” indicates all of the genes that are involved in the entire pathway

### miRNA expression in rat livers treated with furan at a dose of 2 mg/kg bw/day

**Fig. 2:**
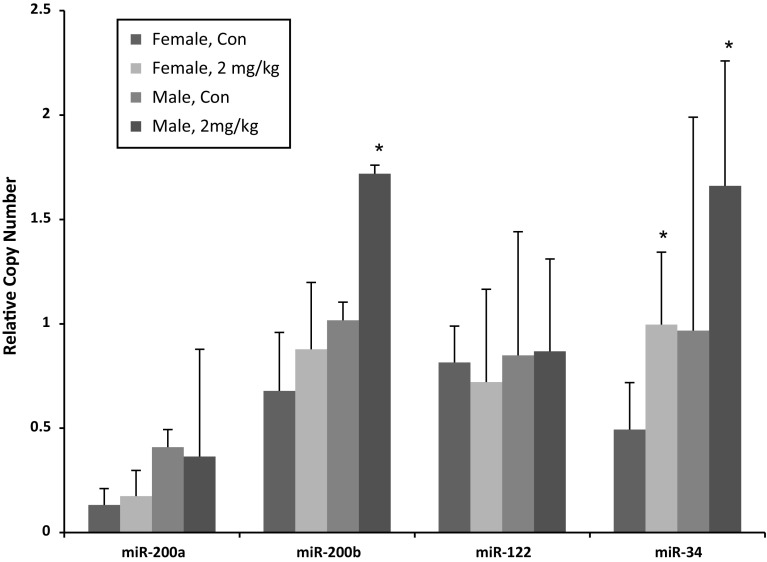
Analysis of miRNA changes using qRT-PCR. RNU6B was used to normalize the expression of the target miRNAs. Three samples from each group were used. Copy number was represented with average ± SD. Experiments were repeated three times and a single representative is shown here. *p ≤ 0.05

Since miRNAs play important roles in the regulation of hepatocellular carcinomas (D’Anzeo et al. 2014) and other biological functions, we examined the effects of furan on miRNA expression at the highest dose in both sexes. Only miR-34a achieved a FDR *p* ≤ 0.05 and absolute FC ≥ 1.5 in either male or female rats based on microarray analyses that showed miR-34a increased by 2.4-fold in both genders. miRNA results were examined in more detail by qRT-PCR for miR-34a and three other miRNAs (miR-122 and miR-200a/b) that were perturbed by 30 mg/kg bw/day furan treatment over 3 months in male *Sprague*-*Dawley* (SD) rats in another study (Chen et al. 2012). qRT-PCR results confirmed the increased expression of miR-34a in both genders and also showed an increase in the expression of miR-200b in male rats only (Fig. 2).

### The effect of furan on TH homeostasis

**Fig. 3:**
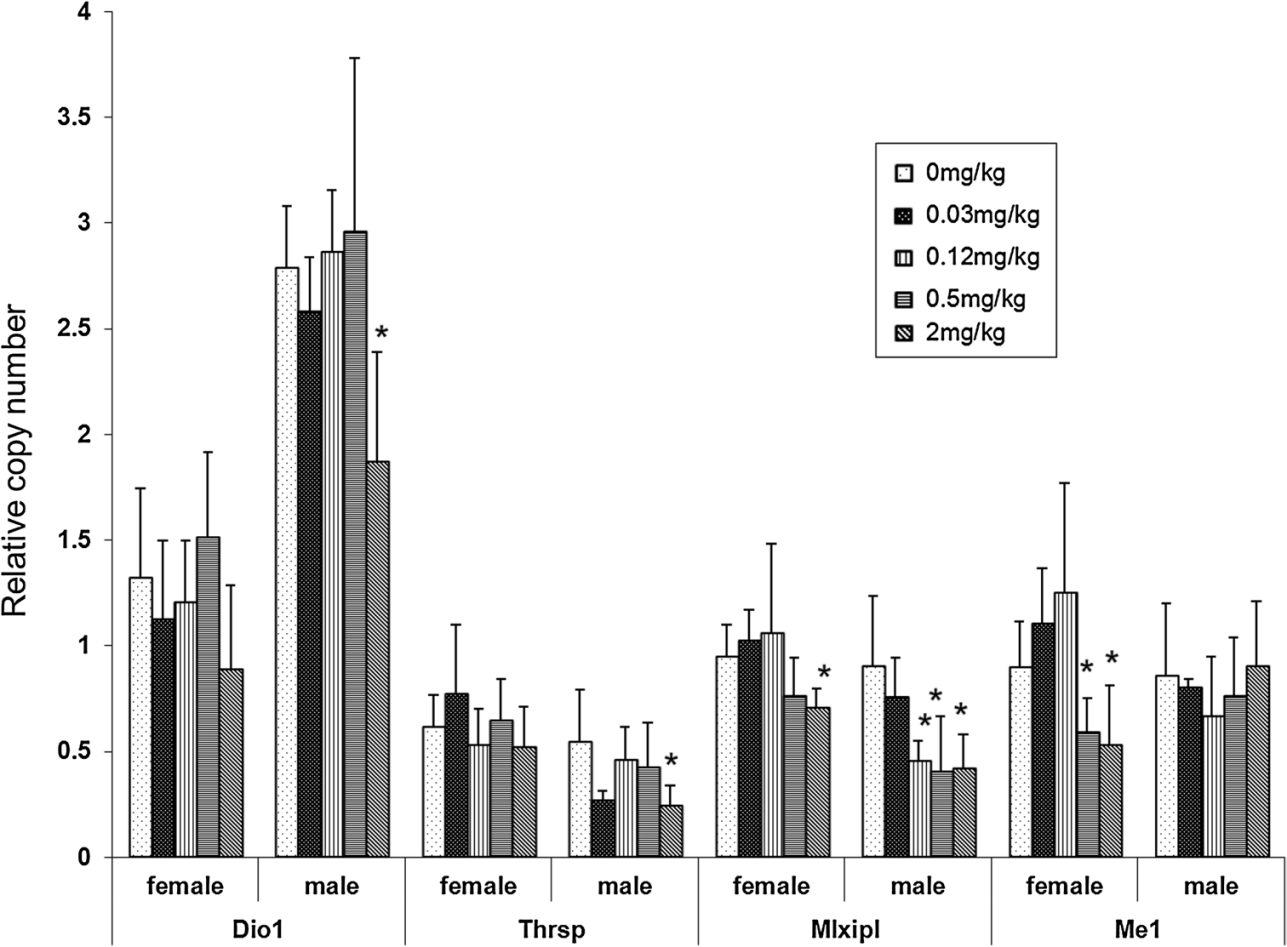
Expression of TH-regulated genes. RNA samples from five female and five male rats in each dose group were examined with qRT-PCR. Each sample was analyzed in duplicate and experiments repeated three times. Representative results are shown. *p ≤ 0.05

Our previous work showed that furan exposure induced a significant increase in serum T4 levels in male rats at doses ≥0.12 mg/kg bw/day, but caused no significant change in female rats (Gill et al. 2010). To further investigate the implications of furan exposure on TH function, we examined the effects of furan on the expression of four genes that are directly regulated by TH in rat liver (*Dio1*, *Thrsp*, *Mlxipl* and *Me1*). *Thrsp* expression in males and *Me1* expression in females were down-regulated, in contrast to the observed increase in serum T4 concentrations in rats treated with 2 mg/kg bw/day furan. qRT-PCR validation of these targets was consistent with the microarray results and also showed significant decreases in the expression of *Dio1* and *Mlxipl* in males in one or more dose groups (Fig. 3). The results indicate that exposure to furan resulted in the down-regulation of TH-responsive genes in both females (*Me1*, *Mlxipl*) and males (*Dio1*, *Thrsp*, *Mlxipl*) despite the observed increase in serum T4 levels only in males.

### Gender differences in response to furan treatment

Analysis of basal gene expression differences between control males and females revealed a total of 958 genes that were significantly different between genders (FC ≥ 1.5; FDR *p* ≤ 0.05; Supplementary Table_S9). There were 594 genes with higher transcript levels in females and 364 lower relative to males. The top three genes that were higher in females were aldo–keto reductase family 1, member B7 (*Akr1b7*: 588-fold higher in females), cytochrome P450, family 2, subfamily c, polypeptide 12 (*Cyp2c12*: 123-fold) and sulfotransferase family 2A, dehydroepiandrosterone (DHEA)-preferring, member 2 (*Sult2a2*: 115-fold). There were 14 transcripts with over a hundred-fold greater levels in males than females including the following: cytochrome P450, family 3, subfamily a, polypeptide 2 (*Cyp3a2*: 768-fold); cytochrome P450, family 4, subfamily a, polypeptide 2 (*Cyp4a2*: 619-fold); alpha-2u globulin PGCL2, PGCL3, PGCL5 and alpha-2u globulin (283-fold, 429-fold, 265-fold and 386-fold, respectively); and several of the major urinary proteins (*Mup4* and *Mup5*; 265-fold and 154-fold, respectively).

Functional annotation clustering of these genes was applied to examine the broad overall trends in the pathways, functions and processes that differed between the sexes (control samples) that might lead to gender-specific responses to furan. The top two functional annotation clusters of genes that were expressed at higher levels in females than in males were involved in lipid and fatty acid metabolism (data not shown). The third cluster was associated with genes involved in xenobiotic metabolism, including significant overrepresentation of alcohol dehydrogenases (e.g., increased expression of *Adh1*, *Adh4*, *Adh5*, *Adh6*, *Adh7*) and genes involved in phase 2 xenobiotic metabolism (e.g., *Gsta5* was 15-fold higher in females than males). In contrast, the top functional clusters that were higher in males than females were also associated with xenobiotic metabolism, but this enrichment accounted for a very large number of phase 1 metabolic genes. In total, of the 19 cytochrome p450s exhibiting different transcript levels between genders, 15 of these were greater in male rat livers. Increased female transcription was only found for *Cyp2c12* (123-fold greater in females), *Cyp26a1* (9-fold greater), *Cypd3a9* (eightfold) and *Cyp2r1* (twofold). There were also some phase 2 genes that were greater in males. In particular, *Gstm1*, *Gstm2*, *Gstm3* and *Gstm4* were all greater in males than females.

**Fig. 4:**
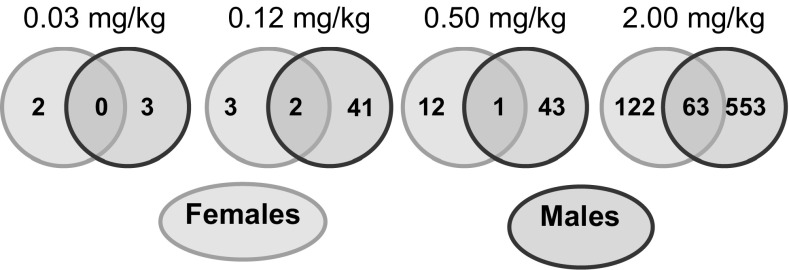
Venn diagram showing the overlap of significantly changed genes (FDR *p* ≤ 0.05 and absolute FC ≥ 1.5) in each dose group between genders

We then explored gender differences in response to the furan treatment. Males exhibited 4–8 times more differentially expressed genes than females in the various treatment groups (Fig. 1a). Only a few genes were similarly perturbed in males and females in the lower-dose groups, while one-third of the genes that were differentially expressed in females were in common with males at 2 mg/kg bw/day (Fig. 4). Cluster analysis of all samples using all significantly changed genes indicated that the samples were distinctly split into two groups determined by sex (supplementary figure_S1).

To further examine gender differences in gene expression, we analyzed the effect of gender using MANOVA in R for the high-dose groups (2 mg/kg bw/day). We first determined differences between the exposed and control groups within gender separately, and then used the combined list to statistically analyze differences between the genders. Setting a cutoff value of FDR *p* ≤ 0.05 and absolute FC ≥ 1.5 between genders, 132 genes that were disregulated by furan treatment exhibited significantly different responses between the genders (supplementary Table_S10). Of these, 86 % of the genes were significantly changed by furan in males only, 10 % were unique to females and the remaining 4 % were altered (but to a different extent) in both genders. The most affected gene that was modulated by a treatment–gender interaction was metallothionein 2A (*Mt2a*), which showed a 9.4-fold increase in response to furan in males relative to controls, but no significant response to furan treatment in females. Interestingly, substantial gender differences in baseline expression levels of most of the genes in the top of the list were observed. These genes had higher basal expression levels in females, and furan treatment up-regulated their expression in males, such as *Cyp2c12* and *Akr1b7*. On the other hand, the genes listed in the bottom of supplementary Table_S10 had higher basal expression in males, and furan exposure down-regulated their expression in males. These results provide potential mechanistic clues for the sensitivities that can be attributed to both differences in basal and induced expression changes.

To further elucidate the mechanisms underlying gender differences following furan treatment, pathway analysis on the 132 genes (supplementary Table_S10) that were different between the genders in the 2 mg/kg bw/day dose groups was conducted. The pathways that responded differently between the genders are shown in Table 6. Differences in the sexes occurred in pathways involved in oxidative stress response and metabolism. Consistent with previous work demonstrating that serum TH levels were altered in male rats alone (Gill et al. 2010), pathway analysis revealed that furan-induced abnormal TH metabolism was impacted by gender. The results were consistent with the pathway analysis that was conducted separately in males and females (Table 2), which indicated that although 65 % of the pathways perturbed in females were also altered in males, the extent was different between genders.

**Table 6:** Pathways demonstrating gender-specific effects in rats treated with 2 mg/kg bw/day furan

Ingenuity canonical pathway	−log (*p* value)	Ratio	Genes
*NRF2*-*mediated oxidative stress response*	2.27	0.03	GSTA3, MGST1, GPX2, GSTM4, GSTP1
*LPS/IL*-*1-mediated inhibition of RXR function*	3.33	0.03	GSTA3, MGST1, LY96, CYP3A7, GSTM4, GSTP1, SULT2A1
*Xenobiotic metabolism signaling*	2.82	0.02	GSTA3, MGST1, UGT2B4, CYP3A7, GSTM4, GSTP1, SULT2A1
*Nicotine degradation*	2.38	0.04	UGT2B4, CYP3A7, Aox3
*Pyrimidine ribonucleotides*	1.89	0.05	NME4, CTPS2
*Thyroid hormone metabolism*	1.77	0.04	UGT2B7, DIO3

−log (*p* value) cutoff at 1.7, ratio indicates the percentage of genes changed significantly in their response to furan between genders to total genes involved in the pathways, genes refer to genes changed significantly in their response to furan between genders

## Discussion

We investigated transcriptional changes occurring in the livers of rats of both sexes treated with furan at the known lowest tested cancer-causing dose and several lower doses for 90 days using DNA microarrays. The transcriptional profiles, pathway perturbations and BMDs provide insight into the MoA and gender differences in response to furan. The data support a similar MoA in rats to that proposed in our previous study in mice for furan-induced hepatocarcinogenesis. BMDs for both apical and transcriptional endpoints associated with this MoA are highly consistent in males. The data reveal that very few miRNAs are perturbed in the liver following subchronic furan exposure in rats. In addition, the effects of furan on TH status are paradoxical.

### Proposed MoA for furan-induced carcinogenicity

Previous global gene expression profiling in our laboratory on female mice exposed to furan for 21 days supported a MoA for hepatocarcinogenesis that involves pathways related to generation of reactive oxygen species causing cytotoxicity, inflammation and regenerative proliferation (Jackson et al. 2014). In the current study on rats, pathway analysis clearly demonstrated induction of an oxidative stress response (NRF2 mediated) and inflammation (LPS/IL-1 mediated) in both genders, although the extent was greater in male rats. The median transcriptional BMD for NRF2-mediated oxidative stress response in males was nearly one half that in females (0.62 vs. 1.12 mg/kg bw/day), supporting that males are more sensitive to furan-induced oxidative stress. These results suggest a similar MoA for furan-induced hepatotoxicity in rats and mice.

NextBio analysis revealed that TAA, nafenopin, MP, mestranol, chloroform, CCl4 and BBZ, all non-genotoxic hepatocarcinogens, exhibited similar gene expression profiles to furan in male rats (supplementary Table_S6). These non-genotoxic hepatocarcinogens act through glutathione depletion and induction of oxidative stress (Uehara et al. 2010), inflammatory response, apoptosis and cell proliferation (Dragan et al. 1996; Fujii et al. 2010; Gill et al. 1998; Golden et al. 1997; Larson et al. 1996). This evidence supports that furan operates through a non-genotoxicity, or indirectly genotoxic, mechanism mediated through oxidative stress to induce hepatocarcinogenesis. Interestingly, Cyp2e1 plays key roles in the metabolism of both TAA and CCl4 to reactive metabolites (Ramaiah et al. 2001), consistent with the metabolic activation of furan by Cyp2e1 (Bakhiya and Appel 2010). Metabolism of Cyp2e1-specific substrates leads to oxidative stress, inflammation and cytotoxicity. Prolonged activation of these processes would cause chronic cytotoxicity potentially leading to hepatocarcinogenesis (Kim et al. 1990; Kim and Novak 1993; Ma et al. 2014; Porter et al. 1989; Song et al. 1986).

The above evidence suggests that a cytotoxicity mechanism mediated through oxidative stress predominates in furan-induced hepatotoxic effects. However, our gene expression data also support a role for genotoxicity in furan-induced hepatotoxicity in males. Specifically (1) P53 was identified as an activated upstream regulator of furan effects (supplementary Table_ S5) in male rats treated with 2 mg/kg bw/day; (2) Ccng1, Fas and Cdkn1a, which all play roles in DNA damage response and other pathways, were significantly up-regulated in male rats at the highest dose; (3) P53 upstream pathways, such as P38 Mapk and Erk Mapk pathways, were among the most sensitive pathways identified using a BMD approach; and (4) miR-34a (P53 target miRNA) is significantly up-regulated. Interestingly, the DNA-damage-inducible gene *Gadd45g* was significantly down-regulated in males at the three highest doses. *Gadd45g* is a tumor suppressor gene that is disrupted in multiple tumor types (Sun et al. 2003; Ying et al. 2005); its down-regulation may have implications in the increased susceptibility of males to cancer.

### Comparison of toxicogenomic and apical BMDs

To further explore the utility of toxicogenomics in predicting adverse effects, we compared BMDs for apical endpoints and transcriptional changes in relevant associated pathways (Table 5). There was insufficient gene expression response to derive pathway BMDs in females; thus, this analysis focussed on males only. The analysis showed a large degree of consistency between apical and transcriptional BMDs in males, and supports the use of transcriptional BMDs for selection of points of departure as we have suggested previously (Bourdon et al. 2012; Jackson et al. 2014). In males, the median transcriptional BMDs for pathways involved in cell damage response ranged from 0.08 to 0.17 mg/kg bw/day, while the BMDs derived from modeling histopathological data on Kupffer cell pigmentation and hepatocyte apoptosis were 0.03 mg/kg bw/day. Kupffer cells are resident macrophages in liver and function as scavengers for cellular debris and blood-born material entering liver. The potential source of the pigment in Kupffer cells seen histologically could be retention of bile, lipofuscin, erythrophagocytosis and disintegrated hepatocytes (Sobaniec-Lotowska and Lebensztejn 2006). Therefore, histological changes, including Kupffer cell pigmentation and hepatocyte apoptosis, could correspond to transcriptional pathways related to cell damage response. The median BMDs for pathways involved in transcriptional inflammatory response were 1.1–1.2 mg/kg bw/day, and the BMD for ALP (an established marker of liver function and inflammation, Browning et al. 2008) was 1.49 mg/kg bw/day. The median transcriptional BMD for bile acid biosynthesis was 1.43 mg/kg bw/day, while analysis of biliary tract hyperplasia and cholangiofibrosis yielded BMDs of 1.45–1.84 mg/kg bw/day, respectively. Bile acid is synthesized in liver, secreted into bile ducts and stored in gallbladder. Bile acids secreted in the bile duct are partly reabsorbed in the cholangiocytes and recycled back to hepatocytes (Chiang 2009). Therefore, abnormal morphology of biliary tract and cholangiocytes (hyperplasia and fibrosis) may affect synthesis and circulation of bile acids. Thus, the BMDs for transcriptional pathways are remarkably aligned with the apical endpoints in males. The results support the use of toxicogenomic data in identifying mechanistically meaningful information to inform hazard characterization and potentially provide effective estimates of points of departure in the absence of apical data (Thomas et al. 2013).

### Effects of furan on miRNA expression

miRNAs are a group of evolutionarily conserved noncoding regulatory RNAs involved in posttranscriptional gene regulation that operate via binding to complementary regions within target transcripts (Bartel 2004). Transcriptional changes in hepatic miRNAs following furan exposure have been studied previously at both low (2 mg/kg bw/day for 4 weeks in male F344 rats) and high doses (30 mg/kg bw/day for 90 days in male SD rats) using PCR arrays (Chen et al. 2010, 2012). These studies identified 5 and 30 miRNAs, respectively, that were significantly changed compared to controls. Similarly, we also found minimal responses in miRNA at 2 mg/kg bw/day of furan. In our study, microarrays detected only a single miRNA significantly changed by furan and this single miRNA was not altered in previous studies on miRNA response to furan (Chen et al. 2010, 2012). Discrepancies may be related to differences in the rat strains (F344 vs. SD), treatment duration (3 months herein vs. 4 weeks) and doses.

The single miRNA significantly changed in our study was miR-34a. It was equally affected in both male and female rats. As a direct target of P53 transcriptional regulation, miR-34a is a well-known tumor suppressor and is involved in apoptosis, cell cycle arrest and senescence (Hermeking 2010). High expression is a negative prognostic factor in human hepatocellular carcinoma (Agostini and Knight 2014) and is associated with abnormal cell growth induced by oxidative stress (Koufaris et al. 2012). The findings provide further evidence to support that the carcinogenicity of furan mainly results from oxidative stress-induced apoptosis.

### The effects of furan on TH homeostasis

Consistent with observed sex-specific differences on gene expression, gender differences were also found in serum TH levels following furan treatment. Increased serum levels of thyroxin (T4, Gill et al. 2010) were observed in males, but not in females. THs are critically important for development and maintenance of metabolic balance in mammals. To determine the biological impact of the increased circulating TH, we examined the effects of furan on the expression of genes related to TH action and metabolism.

Although circulating T4 levels increased following furan exposure in males, the hepatic transcriptional response at least partially indicated a hypothyroid status. Dio1, Thrsp and Mlxipl, well-characterized positive TH-responsive genes in liver, were down-regulated in response to furan. The mechanism(s) underlying this paradox are not clear and were not robustly investigated in this study. An increase in circulating TH is probably due to an increase in the serum levels of carrier proteins (albumin, transthyretic or serpin A7), as the vast majority of circulating T4 is associated with these proteins. Clinical biochemistry data confirm an increase in albumin levels (Gill et al. 2010). We examined the expression of genes involved in TH homeostasis in more detail and found increased expression of *Ugt2b7*, *Dio3*, *Slco1a4* (*Oatp2*) and *Abcb1b* in male rats (supplementary Table_S2, *Slco1a4* significantly increased 1.29-fold). The products of these genes increase TH biliary excretion (Ugt2b7; Szabo et al. 2009; Martin et al. 2012), metabolic deactivation through deiodination (Dio3; Crofton 2008), TH uptake (Slco1a4; Abe et al. 1998) and efflux (Abcb1b; Mitchell et al. 2005; Ribeiro et al. 1996). Therefore, we speculate that increased serum TH levels with a concomitant hypothyroid status in liver tissues may be due to indirect effects of furan exposure (e.g., metabolic enzyme induction) rather than perturbation of the hypothalamic-pituitary-thyroid axis.

### Gender differences in response to low doses of furan

Furan induces liver neoplasms in both male and female rodents (NTP 1993) at doses equal to or >2 mg/kg bw/day. Gender differences have been observed, with higher incidences and lower BMDs for liver neoplasms occurring in male compared to female rodents (Carthew et al. 2010). However, the mechanism(s) underlying gender differences in liver neoplasms induced by furan is (are) largely unexplored. Our study attempted to investigate this issue via dose–response transcriptional analyses in male and female rats.

In general, we observed that the number of significantly changed genes in each dose was about 4–8 times greater in males than females. Overall, male livers were clearly coping with a much higher gene expression response related to oxidative stress (and resulting cytotoxicity) than female livers. Analyzing basal gene expression levels in control males versus females revealed that the top clusters in both sexes related to xenobiotic metabolism (Supplementary Tabel_S9). Males exhibited significantly higher mRNA expression levels of phase I metabolic genes, including 15 cytochrome p450s (19 exhibiting gender differences in total). In contrast, significant overrepresentation of alcohol dehydrogenases (for e.g., increased expression of *Adh1*, *Adh4*, *Adh5*, *Adh6*, *and Adh7*) was found in female livers. The *Adh* family of enzymes provide a general detoxifying system for alcohols, bile acid, aldehydes and lipid peroxidation products. In contrast to the cytochrome P450 system, *Adh* enzymes do not generate toxic radicals during oxidation reactions (Hoog et al. 2001; Westerlund et al. 2007). Thus, high basal levels of *Adh* transcripts may protect female rats from furan toxicity without causing as substantial increases in oxidative stress as occurs in males.

Although there appears to be no differences in severity of histological lesions between males and females at doses ≤2 mg/kg bw/day, there does appear to be a slightly higher frequency of males showing liver pathology at 2 mg/kg bw/day (e.g., biliary tract hyperplasia observed in 6 males vs 1 female) and 0.5 mg/kg bw/day (e.g., basophilic cytoplasm of hepatocytes in 8 males vs 0 females), suggesting increased sensitivity of males (Gill et al. 2010). We found a total of 132 genes that exhibited statistically significant differences between genders (supplementary Table_S10). This can be attributed to the fact that molecular and histological changes are two independent time-related events. Most of these genes had significantly different basal levels of expression between genders. The largest gender differences observed in furan-induced gene expression were metallothionein 2A (*Mt2a*) and glutathione S-transferase Yc2 subunit (*Gsta5*). Among the 132 genes, *Akr1b7* and *Cyp2c12* exhibited the greatest expression differences in the livers of female versus male mice (Kwekel et al. 2010). These genes have some similar features. (1) They all have higher basal expression in females; furan exposure triggered increased expression in males, but levels in males remained lower than or equal to females (data not shown). (2) They all play important roles in detoxification (*Gsta5*, *Akr1b7* and *Cyp2c12*, Endo et al. 2011; Liu et al. 2009; Lushchak 2012; Schmidt et al. 2011), anti-apoptotic effects, anti-oxidative effects and anti-inflammatory actions (*Mt2a*, Endo et al. 2005; Bell and Vallee 2009). Thus, the lower overall overt toxicity observed in females following furan exposure may be due to protective roles executed by these genes. Increased expression of these genes following furan exposure in male rats may indicate that furan triggers these defense systems as a compensatory response.

Pathways were analyzed for all genes that were differently altered by furan treatment in males or females separately, or through statistical analysis of genes that were significantly different through a direct gender comparison of furan-treated rats. Although more than 65 % of the pathways that were perturbed in females were also perturbed in males, statistical significance and number of affected genes within the pathways was consistently lower in females (Table 2). Pathway analysis of genes exhibiting gender differences in response to furan (Table 6) showed that the predominant gender differences were in the NRF2-mediated oxidative stress response and LPS/IL-1 mediated inhibition of RXR function, in each case with effects larger in males than females. Some pathways were exclusively activated in males, including TH metabolism.

Curated disease models with similar gene expression profiles to furan-treated male rats included cancer, inflammatory response and injury of liver (Table 4). In females, disease models included high-fat diet and injury of liver. However, although disease models in females were consistent with males, the correlations between furan exposure and disease models were positive in males, but negative in females. Thus, the expression profiles are virtually inverted for these important disease pathways.

Overall, the observations indicate that while similar toxicological responses are occurring in male and female rats, males are more sensitive (enhanced responsiveness at low doses in key pathways associated with the MoA) to furan toxicity. We speculate that male rats may have progressed further down the disease trajectory to liver cancer at the time of sampling than female rats.

In conclusion, our toxicogenomics data with doses lower or equal to the lowest cancer-causing dose in rats support that furan induces liver carcinogenesis through induction of gene expression pathways related to oxidative stress and inflammatory responses, apoptosis and cellular proliferation. Pathways related to DNA damage response were activated only at the highest (carcinogenic) dose in males, using our stringent FDR-adjusted approach to identification of differentially expressed genes. This approach is useful in delineating the most significant pathways involved in toxicity. However, by modeling the data using a BMD approach, we clearly show that effects associated with pathways involved in cancer are perturbed at doses lower than 2 mg/kg bw/day. Significant gender differences were identified and support that male rats are more sensitive/responsive to furan, consistent with the observed increases in histopathological changes and tumors in male rats. This work is in broad support of the ability of toxicogenomics to provide insight into MoA and gender differences.

## Electronic supplementary material

Below is the link to the electronic supplementary material.

Supplementary material 1 (XLSX 179 kb)

Supplementary material 2 (PPTX 138 kb)
