# Supplementary material for: Toxicogenomic assessment of liver responses following subchronic exposure to furan in Fischer F344 rats
Source: Arch Toxicol. 2015 Jul 21;90:1351–67. doi: 10.1007/s00204-015-1561-2 (PMC4873526; doi:10.1007/s00204-015-1561-2)
Supplement: Supplementary file 2 — Supplementary material 2 (PPTX 138 kb) [file 204_2015_1561_MOESM2_ESM.pptx]

## Slide 1
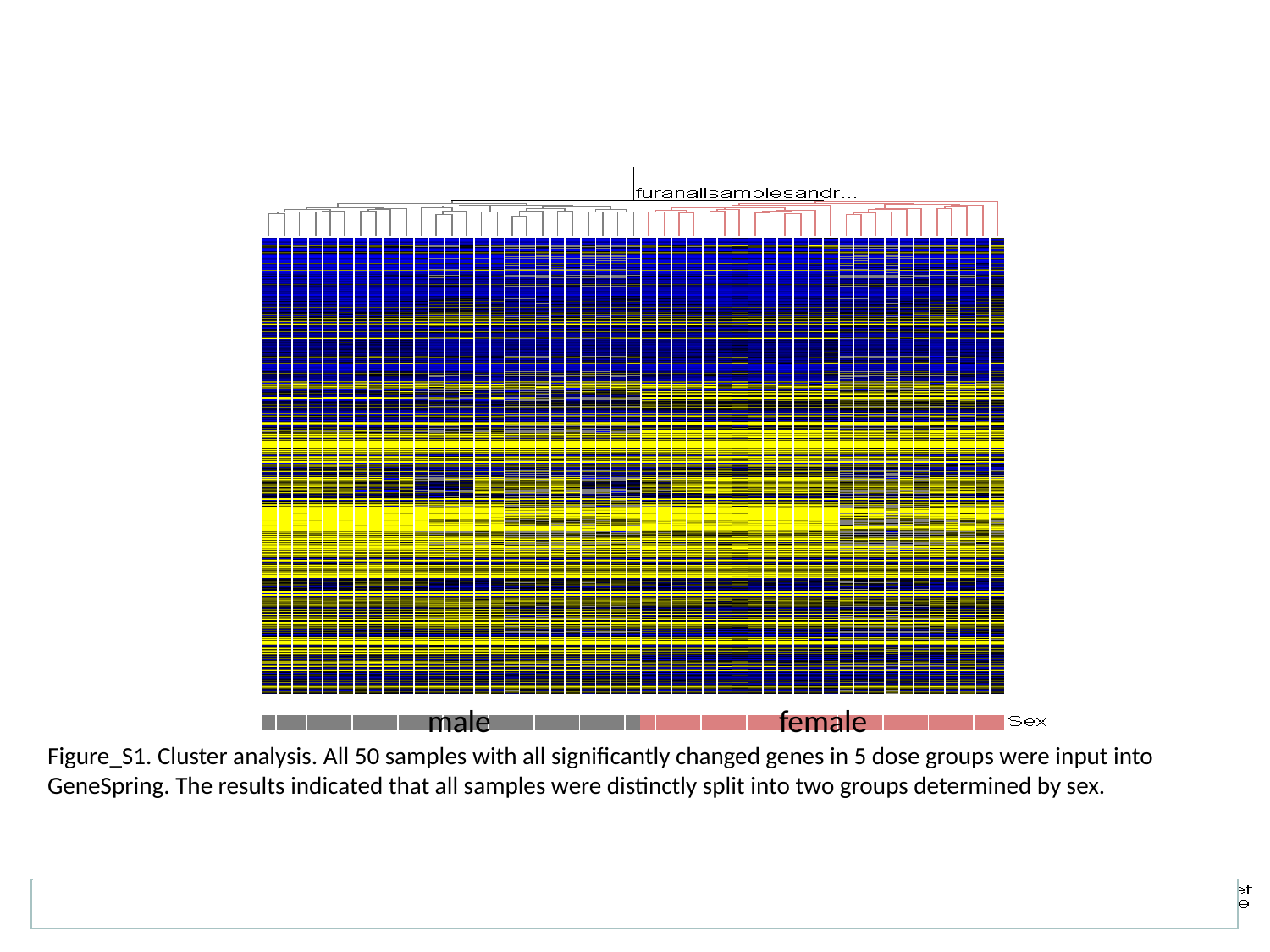

male
female
Figure_S1. Cluster analysis. All 50 samples with all significantly changed genes in 5 dose groups were input into GeneSpring. The results indicated that all samples were distinctly split into two groups determined by sex.
